# Preclinical pharmaco-toxicological screening of biomimetic melanin-like nanoparticles as a potential therapeutic strategy for cutaneous melanoma

**DOI:** 10.3389/fphar.2025.1487854

**Published:** 2025-02-06

**Authors:** Iasmina Marcovici, Raul Chioibas, Istvan Zupko, Iulia Pinzaru, Alina Moaca, Adriana Ledeti, Lucian Barbu-Tudoran, Andreea Geamantan, Iasmina Predescu, Cristina Adriana Dehelean

**Affiliations:** ^1^ Faculty of Pharmacy, “Victor Babes” University of Medicine and Pharmacy from Timisoara, Timisoara, Romania; ^2^ Research Center for Pharmaco-Toxicological Evaluations, Faculty of Pharmacy, “Victor Babes” University of Medicine and Pharmacy from Timisoara, Timisoara, Romania; ^3^ Faculty of Medicine, “Victor Babes” University of Medicine and Pharmacy from Timisoara, Timisoara, Romania; ^4^ CBS Medcom Hospital, Timisoara, Romania; ^5^ Faculty of Pharmacy, University of Szeged, Szeged, Hungary; ^6^ Advanced Instrumental Screening Center, Faculty of Pharmacy, “Victor Babes” University of Medicine and Pharmacy, Timisoara, Romania; ^7^ Electron Microscopy Laboratory “Prof. C. Craciun”, Faculty of Biology and Geology, “Babes-Bolyai” University, Cluj-Napoca, Romania; ^8^ Electron Microscopy Integrated Laboratory, National Institute for R and D of Isotopic and Molecular Technologies, Cluj-Napoca, Romania

**Keywords:** cutaneous melanoma, melanin-like nanoparticles, cytotoxicity, cell migration, epithelial-to-mesenchymal transition, apoptosis, irritant potential, angiogenesis

## Abstract

**Introduction:**

Despite its rarity, cutaneous melanoma (CM) represents the deadliest skin cancer with a high mortality rate, an incidence on the rise, and limited therapeutic options at present. Melanin is a polymeric pigment naturally produced within melanocytes and CM cells that gained a noteworthy attention due to its pharmacological properties, and potential for the design of nanoplatforms with biomedical applications. Up to date, the utilization of melanin-like nanoparticles (MEL-NPs) in cancer treatment has been well-documented, although their efficacy in CM therapy remains scarcely investigated. The current study presents the preclinical evaluation of MEL-NPs as a potential nanomedicine for CM management.

**Methods:**

MEL-NPs were produced through the oxidative polymerization of dopamine and characterized via electron microscopy and UV-VIS spectroscopy. The antioxidant activity was determined by using the DPPH method. The cytotoxic, anti-migratory, anti-clonogenic, pro-oxidant and pro-apoptotic properties of MEL-NPs were investigated *in vitro* by applying the MTT viability test, bright-field and immunofluorescence microscopy, DCFDA/H2DCFDA test, scratch assay, colony formation assay, and RT-qPCR. The irritant and anti-angiogenic effects were assessed *in ovo* on the vascularized chorioallantoic membrane (CAM).

**Results:**

The as-made MEL-NPs presented a spherical morphology, an average size of 85.61 nm, a broad UV-VIS absorption spectrum, and a strong antioxidant activity. After a 24 h treatment, MEL-NPs exerted a selective cytotoxicity in SH-4 and B164A5 CM cells compared to HEMa, HaCaT, and JB6 Cl 41-5a healthy skin cells, except for the concentration of 100 µg/mL, at which their viability declined under 70%. Additionally, MEL-NPs accumulated within the intracellular space of CM cells, forming a perinuclear coating, inhibited their motility and clonogenic potential, increased intracellular oxidative stress, targeted the epithelial-to-mesenchymal transition, and induced apoptosis by altering cell morphology, nuclear aspect, F-actin and tubulin distribution, and by modulating the expression of pro- and anti-apoptotic markers. *In ovo*, MEL-NPs lacked irritant and vascular toxic effects, while exerting an angio-suppressive activity.

**Conclusion:**

MEL-NPs demonstrated promising anti-melanoma properties, showing a selective cytotoxicity, a strong anti-invasive effect and a pro-apoptotic activity in CM cells, while inhibiting CAM angiogenesis, these novel findings contributing to future research on the potential application of this nanoplatform in CM therapy.

## 1 Introduction

Cutaneous melanoma (CM) represents the malignant neoplasm that originates from the cancerous switch of epidermal melanocytes due to aberrant molecular and biochemical changes. Despite being the third most frequent skin malignancy after basal cell and squamous cell carcinomas, accounting for less than 5% of all cases, CM remains the deadliest and the most aggressive cutaneous cancer, having a high metastatic potential and a mortality rate that exceeds 10% (Coricovac et al., 2018; Davey et al., 2021). Epidemiologically, the overall global incidence of CM presents an alarming annual increase, surpassing the rate of any other cancer type (Leonardi et al., 2018). Numerous environmental and genetic risk factors have been correlated with CM oncogenesis (Conforti and Zalaudek, 2021). Although there is a certain interplay between these factors that contributes to CM arising, the excessive exposure to ultraviolet radiation (UVR) emanated by natural or artificial sources remains the greatest trigger for the development of the “cancer that rises with the Sun” (Coricovac et al., 2018; Burns et al., 2019; Davey et al., 2021). Additionally, cutaneous pigmentation–mainly caused by the production and accumulation of melanin (MEL) within the epidermal level of the skin, plays an indisputable key role in the susceptibility of melanocytes to malignant transformation (Conforti and Zalaudek, 2021; Moreiras et al., 2021).

MEL is a generic term defining a widely spread class of pigments with diverse structure and origin, synthesized by melanocytes through a unique and strictly-controlled process known as melanogenesis, via the oxidation and polymerization of tyrosine (Mostert, 2021; Marcovici et al., 2022). This process results in the obtainment of two distinct MEL subtypes: the brown-to-black eumelanin comprising 5,6-dihydroxyindole (DHI) and 5,6-dihydroxyindole-2-carboxylic acid (DHICA) moieties, and the yellow-to-red pheomelanin containing sulphurous benzothiazine and benzothiazole units (Moreiras et al., 2021; Marcovici et al., 2022). Melanogenesis is regulated by several factors acting at the systemic, tissue, cellular, and subcellular levels. UVR was established as the primordial exogenous factor regulating cutaneous melanogenesis, nonetheless, skin pigmentation is also influenced by other factors (e.g., nutritional, endocrine, paracrine, autocrine, intracrine) that involve the interaction between melanocytes and keratinocytes (Slominski et al., 2022). Both melanocytes and CM cells present the ability of producing MEL, and thus causing pigmentation (Bratu et al., 2022). However, melanogenesis is highly dysfunctional in CM cells, which can shift from pigmented to non-pigmented states (Sarna et al., 2019). Therefore, based on the presence or absence of this pigment, CM has been classified as melanotic (pigmented) and amelanotic (unpigmented) (Bratu et al., 2022). Up to date, the ability of MEL to prevent CM development and progression has been documented. The pigment acts as a an UVR-absorbing and photoprotective agent that shields epidermal cells from radiation-induced DNA damage, and oxidative stress, this activity being attributed to the eumelanin subtype (Solano, 2020; Cabaço et al., 2022; Slominski et al., 2022). The presence of endogenous MEL was also correlated to impaired CM metastasis and invasiveness under both *in vitro* and *in vivo* conditions, by enhancing the stiffness and attenuating the elasticity of CM cells, while also diminishing their spread in nude mice (Sarna et al., 2018; 2019; Cabaço et al., 2022). Moreover, amelanotic CM was associated with a lower patient survival rate and higher metastatic potential compared to melanotic CM (Sarna et al., 2019).

Recently, MEL has garnered significant attention from the scientific community owing to its numerous therapeutic properties (e.g., antioxidant, anti-hemolytic, immunomodulatory, anti-neoplastic), exceeding conventional drugs in terms of safety profile and broad activity spectrum (Marcovici et al., 2022). An increasing importance has been appointed to the anti-carcinogenic effect of MEL, its potential efficacy in the treatment of several cancers (e.g., colorectal, leukemia) being explored in previous preclinical studies (Öberg et al., 2009; Al-Obeed et al., 2020; El-Obeid et al., 2020). Additionally, MEL was also found to exert anti-melanoma properties, presenting cytotoxic and pro-apoptotic effects in CM cells (Rudrappa et al., 2023). The abrupt and continuous expansion of nanotechnology, as well as the fascinating functionalities of the natural pigment (e.g., biocompatibility, broadband light absorption, chelating ability, radical-scavenging effects, etc.), fueled the strategic design of versatile MEL-based nanoplatforms for various biomedical applications (Kohri, 2019; Liu et al., 2020; Marcovici et al., 2022). The majority of the as-made MEL-like nanostructures were developed from polydopamine (PDA), the artificial analogue of the natural eumelanin in terms of precursors, synthesis pathways, and structure-property relationship, which has gained a noteworthy popularity especially due to its diverse functions in nanotechnology-based cancer diagnosis and treatment, either as a platform with intrinsic antineoplastic properties, or as a carrier for anti-cancer agents (Liang et al., 2023; Liu et al., 2020; Marcovici et al., 2022). Despite these previous findings, the therapeutic potential of MEL/PDA nanoparticles in CM management remains insufficiently explored nowadays, urging further investigations in this research area.

Therefore, driven by the innate ability of CM in producing MEL, the known implications of the pigment in CM prevention, the inherent anti-melanoma effects of MEL, the unprecedented outburst in MEL/PDA-based nanotechnological advancements and the recent studies of the anti-tumor activity of MEL and MEL/PDA nanoparticles, the present study endeavors to conduct a preclinical evaluation of the anti-cancer properties retained by artificially produced bioinspired MEL nanoparticles (MEL-NPs) as a potential platform with applications in CM therapy. Debuting with the physicochemical characterization of the obtained MEL-NPs, the study continues by exploring their safety profile in healthy cutaneous cells (human melanocytes - HEMa, human keratinocytes - HaCaT, murine epidermal cells - JB6 Cl 41-5a) and anti-neoplastic properties in CM cells (human melanoma cells - SH-4, murine melanoma cells - B164A5) and ends with the investigation of their irritant potential and anti-angiogenic effect *in ovo*, furnishing novel perspectives to the applications of MEL-like nanostructures in CM treatment.

## 2 Materials and methods

### 2.1 Reagents, cell lines and instruments

The following reagents were received from Thermo Fisher Scientific Inc., (Waltham, MA, United States): dopamine (3-hydroxythyramine) hydrochloride, ultrapure distilled water, alpha tubulin monoclonal antibody (B-5-1-2), Texas Red™-X phalloidin, goat anti-mouse IgG (H+L) secondary antibody (Alexa Fluor™ 488), Invitrogen™ PureLink™ RNA Mini Kit, 10x DNase I reaction buffer, DNase I Amplification Grade and Power SYBR™ Green RNA-to-CT™ 1-Step Kit. The MTT [3-(4,5-dimethylthiazol2-yl)-2,5-diphenyltetrazolium bromide] assay kit, DAPI (4′,6-diamino-2-phenylindole), diphenylpikrylhydrazyl (DPPH), TritonX-100, penicillin/streptomycin solution, phosphate saline buffer (PBS), sodium lauryl sulfate (SLS), the B164A5 cell line, and ascorbic acid were bought from Sigma Aldrich, Merck KgaA (Darmstadt, Germany). Dulbecco’s Modified Eagle Medium (DMEM; 30–2002™), Eagle’s Minimum Essential Medium (EMEM; 30-2003™), Dermal Cell Basal Medium, Adult Melanocyte Growth Kit, Penicillin-Streptomycin-Amphotericin B solution, dimethyl sulfoxide (DMSO), fetal bovine serum (FBS), Trypsin-EDTA solution, and HEMa (PCS-200-013™), JB6 Cl 41-5a (CRL-2010™), and SH-4 (CRL-7724™) cells were acquired from ATCC (American Type Culture Collection, Lomianki, Poland). Sodium hydroxide was obtained from Honeywell International Inc. (Charlotte, North Carolina, United States). Crystal violet 1% was bought from Electron Microscopy Sciences (Hatfield, PA, United States). Paraformaldehyde 4% was delivered by Santa Cruz Biotechnology (Dallas, TX, United States). DCFDA/H2DCFDA–Cellular ROS Assay Kit was provided by Abcam (Cambridge, United Kingdom). The Lionheart FX microscope, the Cytation 5 microplate reader, the AutoScratch™ Wound Making Tool, and the Gen5™ Microplate Data Collection and Analysis Software (Version 3.14) were provided by BioTek Instruments Inc., Winooski, VT, United States.

### 2.2 MEL-NPs synthesis method

MEL-NPs were synthesized through the spontaneous oxidation and polymerization of dopamine in alkaline conditions by applying a protocol similar to the one presented by Li et al. (2018). Simply, dopamine hydrochloride was dissolved in ultrapure sterile distilled water and treated with a solution of NaOH 1 M. The color of the solution turned yellow after alkalinization and gradually changed to brown. After stirring for 5 h at 50°C, the black granular MEL-NPs were retrieved through high-speed centrifugation (15,000 rpm, 20 min), washed, and redispersed in ultrapure sterile distilled water. The obtained MEL-NPs were stored at 4°C until further use.

### 2.3 Electron microscopy and UV-VIS spectroscopy analyses

The size and morphology of MEL-NPs were determined through scanning electron microscopy (SEM) and transmission electron microscopy (TEM), respectively, using the microscope Hitachi SU8230 cold field emission gun STEM (Chiyoda, Tokyo, Japan) equipped with EDX detectors X-MaxN 80 (Oxford Instruments, United Kingdom) and following the procedure reported previously by Moacă et al. (2023). The UV-VIS spectrum of MEL-NPs was obtained using the Jena Analytik Specord 250 Plus double beam spectrophotometer (Jena, Germany) with matched quartz cells of 1 cm, at a wavelength range between 200 and 1,000 nm.

### 2.4 Antioxidant activity evaluation

The antioxidant potential of the obtained MEL-NPs was assessed by evaluating their ability to scavenge the DPPH free radical, as presented previously by Mansour et al. (2016). Thus, 50 µL of samples (MEL-NPs) and 150 µL of DPPH methanol solution were added in a 96-well plate. The concentration of MEL-NPs was maintained at 10, 25, 50, 75, and 100 µg/mL, respectively, while the DPPH concentration in each well was 200 µM. Next, the plate was incubated at room temperature, protected from light for 20 min and the absorbance was read at 520 nm using Cytation 5. Ascorbic acid (10, 25, 50, 75, and 100 µg/mL) was selected as a positive control. The antioxidant activity (%) was calculated as follows:

$$\text{Antioxidant activity }\left(\%\right)=\frac{\left(AbsorbanceDPPH-AbsorbanceSample\right)}{AbsorbanceDPPH}\;x\;100$$

### 2.5 Cell culture protocol

The cell lines selected for the present study were cultured in standard conditions–humidified atmosphere, 37°C, and 5% CO_2_. All procedures were performed following the manufacturers’ protocols. Briefly, HaCaT, SH-4, and B164A5 cells were grown in DMEM with 10% FBS, JB6 Cl 41-5a cells in EMEM with 5% FBS, and HEMa in Dermal Cell Basal Medium containing one Adult Melanocyte Growth Kit. DMEM and EMEM were supplemented with 1% Penicillin-Streptomycin mixture, while 500 µL of Penicillin-Streptomycin-Amphotericin B Solution were added to the Dermal Cell Basal Medium. The cells presented normal morphology, growth and proliferation during the experiments.

### 2.6 Cytotoxicity assessment

The cytotoxic effect of MEL-NPs on HEMa, HaCaT, JB6 Cl 41-5a, SH-4 and B164A5 cells was assessed by applying the MTT viability assay after 24 h of treatment. In brief, at the end of the incubation interval, 10 µL of MTT reagent were added in every well, and the plates were incubated for 3 h at 37°C and 5% CO_2_. Finally, 100 µL of MTT solubilizing solution were added, the plates were kept at room temperature for 30 min, in the dark, and the absorbance was measured on Cytation 5 at two wavelengths - 570 and 630 nm.

### 2.7 Cell morphology and intracellular distribution of MEL-NPs

The localization of the obtained MEL-NPs within SH-4 and B164A5 cells was assessed microscopically. Representative images were taken using the Lionheart FX microscope and the images were analyzed in bright-field using the Gen5™ Microplate Data Collection and Analysis Software Version 3.14.

### 2.8 Immunofluorescence staining of nuclei, tubulin and F-actin

The impact of the 24 h treatment with MEL-NPs on nuclear morphology and the distribution of cytoskeletal F-actin and tubulin filaments was investigated through immunofluorescence staining. Initially, the cells were grown in clear-bottom, black 96-well plates, left to attach, and exposed to MEL-NPs for the indicated period of time. Then, they were treated with paraformaldehyde 4%, TritonX 0.1%, and bovine serum albumin (BSA) 1%. Tubulin was visualized by exposing the cells to the alpha tubulin monoclonal antibody (B-5-1-2) for 4 h and the goat anti-mouse IgG (H+L) secondary antibody (Alexa Fluor™ 488) for 45 min. F-actin was stained with Texas Red™-X phalloidin for 30 min at room temperature. The cell nuclei were counterstained with a DAPI solution for 5 min. The treatments were performed at room temperature and protected from light. These steps were also preceded by the plates’ three-times washing with PBS. At the end, the cells were imaged using the Lionheart FX microscope and analyzed in the Gen5™ Microplate Data Collection and Analysis Software Version 3.14. The apoptotic index was finally calculated by applying the following formula:

$$\text{Apoptotic index }\left(\%\right)=\frac{Number\;of\;apoptotic\;nuclei}{Total\;number\;of\;nuclei}\;x\;100$$

### 2.9 Measurement of intracellular oxidative stress

The ability of MEL-NPs to trigger oxidative stress in SH-4 and B164A5 cells was determined by applying the DCFDA/H2DCFDA–Cellular ROS Assay Kit according to the protocol provided by the manufacturer. For this experiment, the cells were cultured in clear-bottom, black 96-well plates, left to adhere, and treated with MEL-NPs for 24 h. Next, the culture medium was removed, the cells were washed with 1X Buffer and treated for 45 min at 37°C and 5% CO_2_ with a DCFDA solution prepared in 1X Buffer at a concentration of 20 µM. Finally, the DCFDA solution was removed and 1X Supplemented Buffer was added. The volume used in these steps was 100 µL/well. Representative images were taken using the Lionheart FX microscope and fluorescence was measured at excitation/emission of 485/535 nm on Cytation 5. The images and fluorescence data were analyzed in the Gen5™ Microplate Data Collection and Analysis Software Version 3.14.

### 2.10 Automatic scratch assay for cell migration

The influence of MEL-NPs on the migration of SH-4 and B164A5 cells was assessed by performing the automatic scratch assay. In brief, the cells were cultured in Corning Costar 24-well plates, an automatic scratch was made in each well using the AutoScratch™ Wound Making Tool, the cells were treated with MEL-NPs for 24 h, and representative images (at magnification ×4) of the scratch area were taken at 0 and 24 h post-treatment on Cytation 1 (BioTek^®^ Instruments Inc., Winooski, VT, United States). The width of the performed scratches was measured in Gen5 ™ Microplate Data Collection and Analysis Software Version 3.14, and the migration rates were calculated using the formula (Simu et al., 2021):

$$\text{Migration rate }\left(\%\right)=\frac{At0-At24}{At0}\;x\;100,\text{where}$$

At0 = scratch width at 0 h.At24 = scratch width at 24 h.

### 2.11 Colony formation test

The ability of MEL-NPs (10 µg/mL) to inhibit colony formation in SH-4 and B164A5 cells was assessed through the colony formation test. Shortly, the cells were cultured in 6-well plates at a low density of 400 cells/well and left to attach. Then, the cells were treated with MEL-NPs for 24 h, and the medium was changed regularly with fresh one for 7–10 days. At the end of the experiment, the cells were fixed with paraformaldehyde 4% and stained for 10 min at room temperature with crystal violet 0.2% diluted in PBS. Following the rinsing of the wells with water and acquisition of representative images, the cells were lysed with SLS 1%, and the absorbance was read at 550 nm on Cytation 5. The number of formed colonies was determined microscopically. A similar method was described by Han et al. (2018).

### 2.12 RT-qPCR analysis of mRNA expressions

For this assay, SH-4 CM cells were grown in 6-well plates at a density of 10^6^ cells/well and treated with MEL-NPs for 24 h. RNA was extracted using the Invitrogen™ PureLink™ RNA Mini Kit by following the manufacturer’s instructions and quantified using the DS-11 spectrophotometer (DeNovix, Wilmington, DE, United States) at 260/280 nm. The contamination with genomic DNA was eliminated by subjecting all RNA samples to a treatment with DNase I. To accomplish this, the RNA samples were incubated for 15 min at room temperature 10X DNase I Reaction Buffer and DNase I Amplification Grade, further treated with 25 mM EDTA for DNase I inactivation and heated for 10 min at 65°C. Reverse-transcription was conducted using the Power SYBR™ Green RNA-to-CT™ 1-Step Kit according to the protocol available from the manufacturer. Each reaction contained sample RNA, Power SYBR^®^ Green RT-PCR Mix (2x), RT Enzyme Mix (125x), specific primers (forward and reverse), and RNase-free water at a final volume of 20 µL. The reaction plate was sealed with optical adhesive film and cDNA amplification was performed on the Quant Studio 5 real-time PCR system (Thermo Fisher Scientific, Inc., Waltham, MA, United States) using the following thermal conditions: 30 min at 48°C, 10 min at 95°C, 40 cycles at 95°C (15 s), and 1 min at 60°C, as recommended by manufacturer of the kit. Relative mRNA expressions were determined by applying the 2^−ΔΔCT^ method, normalized to β-actin used as housekeeping gene and further expressed as fold change relative to control. The used forward and reverse primer sequences (Table 1) were specifically designed to target the respective genes and commercially provided by Eurogentec (Seraing, Belgium) and Invitrogen (Waltham, MA, United States).

**TABLE 1:** Forward and reverse sequences of the gene-specific primers used in this study.

Primer	Forward	Reverse
β-actin	5′AGA​GGG​AAA​TCG​TGC​GTG​AC3′	5′CAA​TAG​TGA​TGA​CCT​GGC​CGT3′
E-cadherin	5′TTC​CTC​CCA​ATA​CAT​CTC​CC3′	5′TTG​ATT​TTG​TAG​TCA​CCC​ACC3′
Vimentin	5′CTC​TTC​CAA​ACT​TTT​CCT​CCC3′	5′AGT​TTC​GTT​GAT​AAC​CTG​TCC3′
MMP-2	5′ATG​ACA​GCT​GCA​CCA​CTG​AG3′	5′ATT​TGT​TGC​CCA​GGA​AAG​TG3′
MMP-9	5′TTG​ACA​GCG​ACA​AGA​AGT​GG3′	5′GCC​ATT​CAC​GTC​GTC​CTT​AT3′
Bad	5′CCC​AGA​GTT​TGA​GCC​GAG​TG3′	5′CCCATCCCTTCGTCCT3′
Bak	5′ATG​GTC​ACC​TTA​CCT​CTG​CAA3′	5′TCA​TAG​CGT​CGG​TTG​ATG​TCG3′
Bax	5′GCC​GGG​TTG​TCG​CCC​TTT​T3′	5′CCG​CTC​CCG​GAG​GAA​GTC​CA3′
Bcl-XL	5′GAT​CCC​CAT​GGC​AGC​AGT​AAA​GCA​AG3′	5′CCC​CAT​CCC​GGA​AGA​GTT​CAT​TCA​CT3′

### 2.13 *In ovo* experimentation on the chorioallantoic membrane (CAM)

The *in ovo* experiments were performed using white fertilized chicken eggs (Gallus *gallus domesticus*), as previously described (Rednic et al., 2022). On the first day of receiving, the eggs were washed, disinfected with 70% alcohol, and carefully transferred to the incubator. On the fourth day of incubation, a cut was made at the tip of the egg, allowing the extraction of approximately 6–7 mL of albumen. The next day of incubation, a small window was cut in the upper part of each egg and then covered with adhesive tape. The eggs were placed back into the incubator, and kept in standard conditions (37°C, 60% humidity) during the experiments which were finalized before reaching the 14th day of embryonic development.

### 2.14 Hen’s egg CAM irritation test

The HET-CAM test was performed at the 10th day of eggs’ incubation, following a procedure described by Kis et al. (2022). The potential irritant effect of MEL-NPs at concentrations of 10, 50, and 100 µg/mL was compared with the one exerted by SLS 1% used as positive control and distilled water (H_2_O) used as negative control. Briefly, a volume of 600 µL of positive control, negative control and sample was applied to the CAM. Signs of hemorrhage (H), lysis (L) and vascular coagulation (C) were observed for 5 min. Representative photographs of the CAM were taken before the application of the samples (at T0) and 5 min after their application (at T5). The vascular changes were observed and evaluated using a stereomicroscope (Stereomicroscope Discovery 8; Zeiss). The images were taken with color Axio CAM 105-Zeiss and then processed using the ZEN core version 3.8 software. The possible irritating potential of MEL-NPs was determined using the irritation scoring method which classifies substances based on the irritation score (IS) value as non-irritating (IS = 0–0.9), irritating (IS = 1–8.9) and severely irritating (IS = 9–21) (Racea et al., 2023).

### 2.15 *In ovo* angiogenesis study

The potential angio-inhibitory effect of MEL-NPs was assessed using chicken fertilized eggs at the 8–10th day of embryonic development to cover the period at which the CAM reaches the highest point of neovascularization (Aventurado et al., 2020). The applied method was presented in a previous study (Parveen and Nadumane, 2020). Shortly, MEL-NPs were directly applied on the CAM, the eggs were incubated for a period of 24 h, and representative images were obtained using the Discovery 8 SteREO microscope and the ZEN core version 3.8 software. The quantitative analysis of the vascular area and number of branching points was conducted on the IKOSA Prism Application CAM assay (Version 3.1.0).

### 2.16 Statistical analysis of the results

The data from this paper are presented as means ± standard deviation (SD). The differences between data were compared in GraphPad Prism 10 version for Windows (GraphPad Software, San Diego, CA, United States, www.graphpad.com), using the one-way ANOVA analysis, the Dunett’s multiple comparisons post-test, and the unpaired t-test. Statistical significance was marked with * (*p < 0.05; **p < 0.01; ***p < 0.001; ****p < 0.0001 versus Control), respectively.

## 3 Results

### 3.1 Characterization of MEL-NPs

**FIGURE 1:**
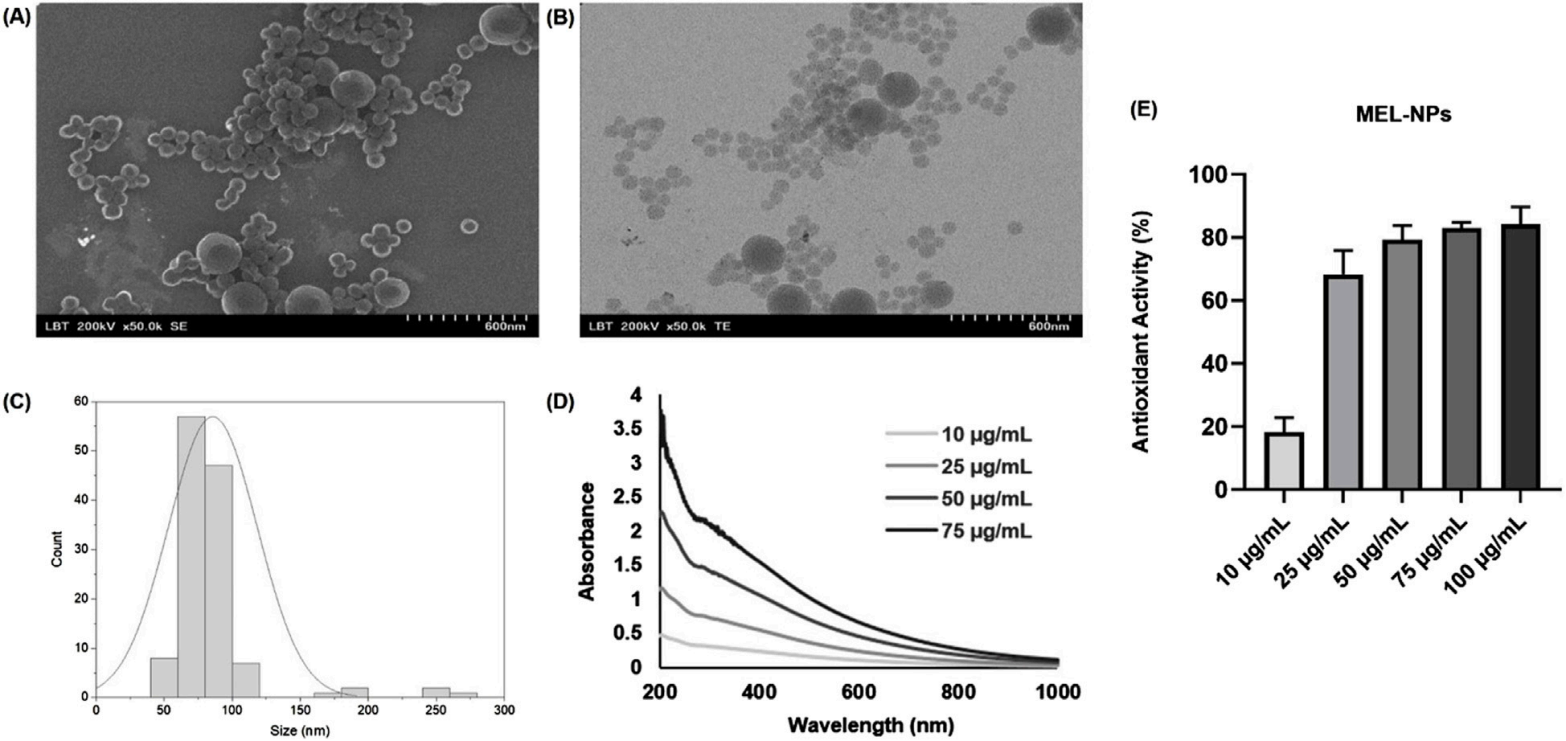
**(A)** Scanning electron microscopy (SEM) image, **(B)** Transmission electron microscopy (TEM) image, **(C)** Size distribution histogram, **(D)** UV-VIS spectrum, and **(E)** Antioxidant activity of MEL-NPs.

Figure 1 depicts the characterization of the synthesized MEL-NPs that presented a well-defined spherical shape and an average size of 85.61 nm although a small population of MEL-NPs showed a dimension that was over 100 nm. Four samples were evaluated by UV-VIS spectrophotometry at different concentrations of MEL-NPs (10, 25, 50 and 75 μg/mL). A strong relationship between the concentration of MEL-NPs and the absorbance was found, as its values increased with the increase of the MEL-NPs concentration. MEL-NPs presented a monotonic and broad-band absorption in the UV-VIS spectrum, the absorbance gradually decreasing from 200 nm to 1,000 nm at all tested concentrations. The antioxidant potential of MEL-NPs at five concentrations of interest – 10, 25, 50, 75, and 100 µg/mL–was evaluated using the DPPH assay. The ability of MEL-NPs to scavenge the DPPH free radical increased with increasing concentrations. Thus, at 10 µg/mL, the antioxidant activity was 18.27%, and gradually reached 68.31%, 79.27%, 83.03%, and 84.17% at higher concentrations (25, 50, 75, and 100 µg/mL, respectively). The antioxidant property of MEL-NPs was slightly lower compared to ascorbic acid used as positive control, which exerted a dose-dependent efficacy in scavenging the DPPH radical at these concentrations, the highest antioxidant activity (96.11%) being reached at 100 µg/mL (data not shown). The estimated IC_50_ for MEL-NPs, representing the concentration at which 50% of the DPPH radical was scavenged, is 20.38 µg/mL.

### 3.2 Cytocidal potential of MEL-NPs in healthy and CM cells

**FIGURE 2:**
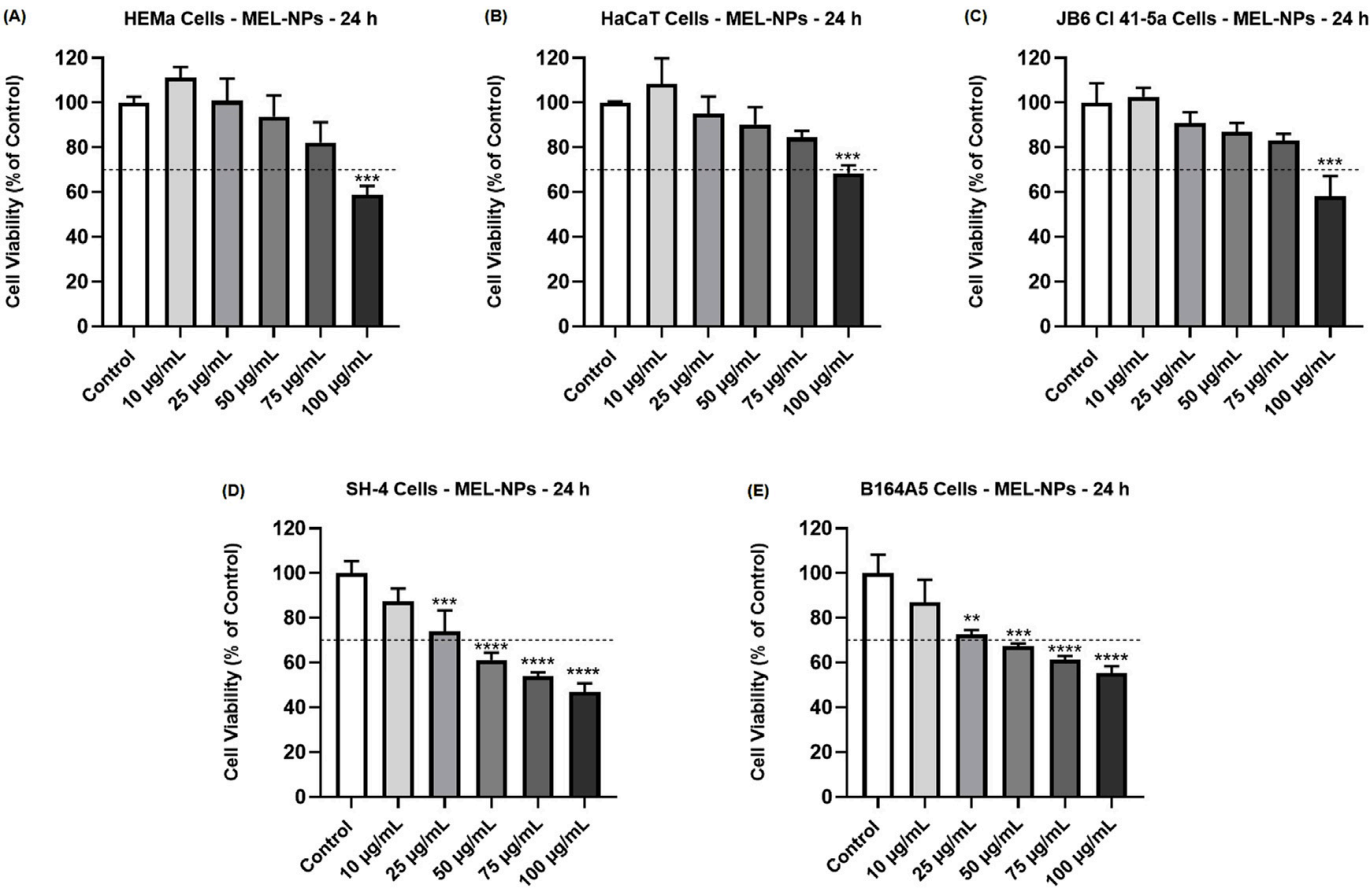
Influence of MEL-NPs (10, 25, 50, 75 and 100 µg/mL) on the viability of **(A)** HEMa, **(B)** HaCaT, **(C)** JB6 Cl 41-5a, **(D)** SH-4 and **(E)** B164A5 cells following a 24 h treatment. The data were normalized to control (representing cells without treatment) and expressed as means ± SD of three independent experiments conducted in triplicate. The statistical differences between Control and MEL-NPs-treated groups were determined using the one-way ANOVA analysis and the Dunnett’s multiple comparisons post-test (**p < 0.01; ***p < 0.001; ****p < 0.0001 versus control). Horizontal line at 70% indicates the viability percentage below which a cytotoxic potential is considered.

The cytotoxicity of MEL-NPs in healthy skin-derived cells (HEMa, HaCaT, JB6 Cl 41-5a) and CM cells (SH-4, B164A5) was assessed by evaluating their impact on cell viability after a 24 h treatment (Figure 2). MEL-NPs significantly lowered the viability of HEMa, HaCaT, and JB6 Cl 41-5a cells only at the highest concentration of 100 µg/mL (to 58.89%, 68.43%, and 58.14%, respectively). At 10 µg/mL, MEL-NPs exerted a slight stimulatory effect on the cells’ viability which increased to 111.14% (HEMa), 105.74% (HaCaT) and 102.53% (JB6 Cl 41-5a), while at 25, 50, and 75 µg/mL, it was maintained over 80% in all healthy cell lines. Comparatively, the 24 h treatment of the CM cells with MEL-NPs caused a concentration-dependent decline in cell viability that reached statistical significance starting with the concentration of 25 µg/mL. The viability of SH-4 cells was gradually reduced by MEL-NPs from 87.27% (at 10 µg/mL) to 73.98% (at 25 µg/mL), 61.12% (at 50 µg/mL), 53.98% (at 75 µg/mL), and 46.81% (at 100 µg/mL), respectively. The percentage of viable B164A5 cells was also lowered by the 24 h exposure to MEL-NPs to 86.95% (at 10 µg/mL), 72.66% (at 25 µg/mL), 67.30% (at 50 µg/mL), 61.23% (at 75 µg/mL), and 55.24% (at 100 µg/mL), respectively. Cytotoxicity, suggested by a reduction in cell viability below 70%, was observed at 100 µg/mL in the case of HEMa, HaCaT and JB6 Cl 41-5a healthy cells, as well as starting with the concentration of 50 µg/mL in the case of SH-4 and B164A5 CM cells.

### 3.3 Cell morphology and intracellular localization of MEL-NPs

**FIGURE 3:**
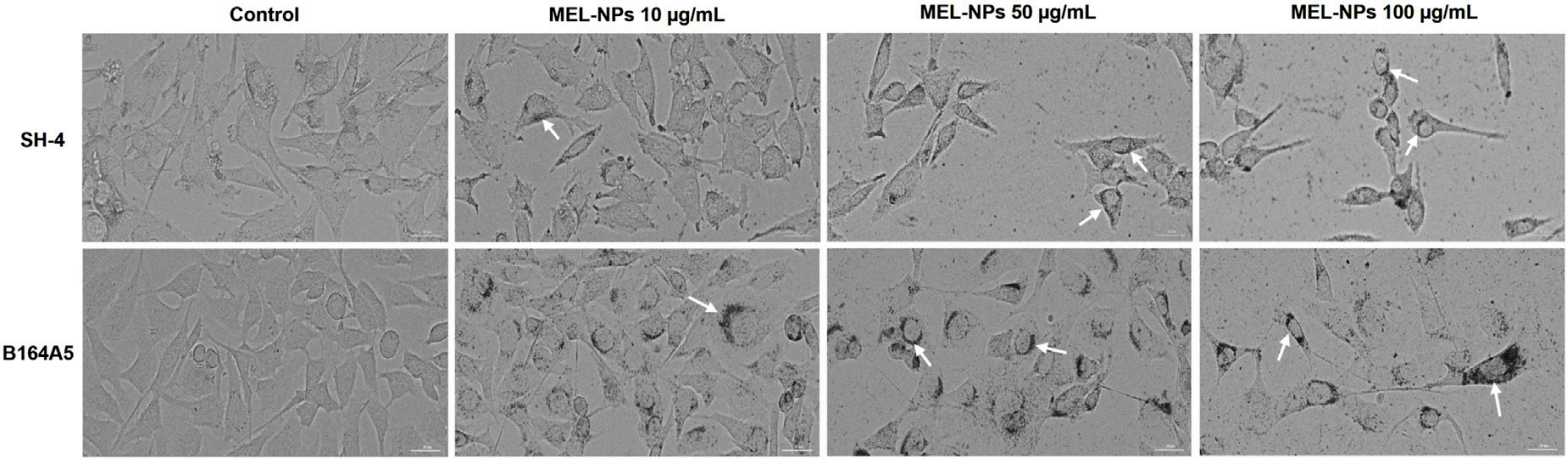
Representative images illustrating the morphology and confluence of SH-4 and B164A5 cutaneous melanoma (CM) cells treated with MEL-NPs (10, 50, and 100 µg/mL) for 24 h. The arrows indicate the presence of MEL-NPs within the intracellular space. The scale bars indicate 30 µm.

To explore the intracellular localization of MEL-NPs and their impact on cellular morphology and confluence, the aspect of SH-4 and B164A5 cells following treatment was further observed (Figure 3). After 24 h of treatment, MEL-NPs efficiently infiltrated within the SH-4 cells’ cytoplasmic space, accumulating in the perinuclear area (white arrows) at all tested concentrations, however, the highest amount of internalized MEL-NPs was obtained at 100 µg/mL. This effect was associated with a concentration-dependent loss of cell confluence and changes in the cells’ morphology which adopted a spherical shape and a reduced size compared to control. A similar behavior was also noticed in B164A5 cells, although a higher intracellular accumulation was detected, MEL-NPs forming a more prominent perinuclear cap in this cell line compared to SH-4 cells. In comparison to control, cell rounding, shrinkage, and loss of confluence were observed in B164A5 cells treated with MEL-NPs (10, 50, and 100 µg/mL) for 24 h.

### 3.4 Impact of MEL-NPs on cell nuclei and cytoskeletal filaments

**FIGURE 4:**
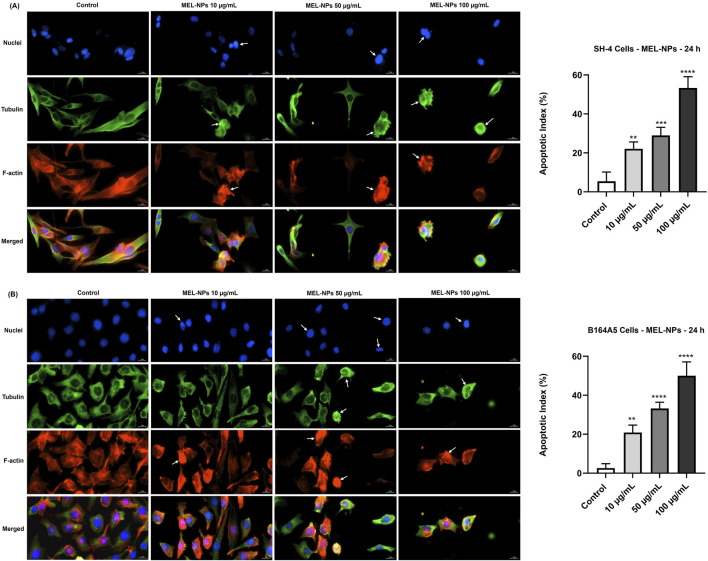
**(A)** Representative images of SH-4 cells’ nuclei, F-actin, and tubulin filaments and apoptotic index percentages after a 24 h treatment with MEL-NPs (10, 50, and 100 µg/mL) and **(B)** Representative images of B164A5 cells’ nuclei, F-actin, and tubulin filaments and apoptotic index percentages after a 24 h treatment with MEL-NPs (10, 50, and 100 µg/mL). The scale bars indicate 20 μm, and the arrows indicate apoptotic-like changes in the aspect of nuclei, tubulin and F-actin. The data are expressed as means ± SD of three independent experiments conducted in triplicate. The statistical differences between Control and MEL-NPs-treated groups were determined using the one-way ANOVA analysis and the Dunnett’s multiple comparisons post-test (**p < 0.01; ***p < 0.001; ****p < 0.0001 versus control).

The potential changes in the aspect of cellular components (nuclei, tubulin, and F-actin) following the CM cells’ treatment with MEL-NPs was next investigated (Figure 4). MEL-NPs caused visible alterations in SH-4 cells after 24 h, evidenced by a considerable constriction of both cell nuclei and cytoskeletal tubulin and F-actin fibers associated with cell rounding, as well as by a significant increase in apoptotic index (over 20%) at all tested concentrations. Additionally, at 50 and 100 µg/mL, the SH-4 cells exposed to MEL-NPs presented signs of bleb formation. MEL-NPs also induced several changes in the aspect of B164A5 cells’ nuclei, tubulin and F-actin at all tested concentrations after 24 h of treatment. At 10 µg/mL, a slight chromatin constriction but an intense condensation of F-actin at the peripheral area of the cells or within the entire cell space were observed, while tubulin maintained a distribution similar to control. At 50 and 100 µg/mL, all cellular components appeared massively condensed whereas the cells became spherical in shape. The size of the nuclei and the longitudinal axis of the cells exposed to MEL-NPs were visibly reduced compared to control. A significant elevation in apoptotic index was registered in B164A5 cells treated for 24 h with MEL-NPs (10, 50, and 100 µg/mL).

### 3.5 Effect of MEL-NPs on intracellular oxidative stress

**FIGURE 5:**
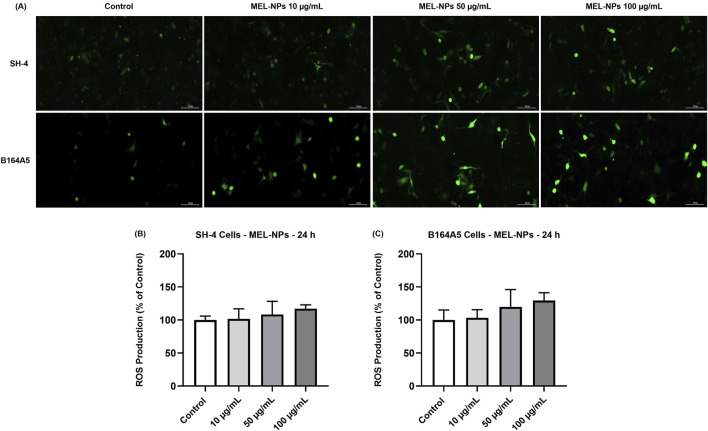
**(A)** ROS generation in SH-4 and B164A5 cutaneous melanoma (CM) cells observed under a fluorescence microscope at ×20 magnification after a 24 h treatment with MEL-NPs (10, 50, and 100 µg/mL). The scale bars indicate 100 µm. Measurement of ROS in **(B)** SH-4 cells and **(C)** B164A5 cells following a 24 h treatment with MEL-NPs (10, 50, and 100 µg/mL). The data were normalized to control (representing cells without treatment) and expressed as means ± SD of three independent experiments conducted in triplicate. The statistical differences between Control and MEL-NPs-treated groups were determined using the one-way ANOVA analysis and the Dunnett’s multiple comparisons post-test.

The potential impact of MEL-NPs on the oxidative stress status in SH-4 and B164A5 CM cells was next investigated (Figure 5). The results indicated a concentration-dependent increase in ROS production in both cell lines after 24 h of treatment. In SH-4 cells the percentages (101.54% at 10 µg/mL, 108.32% at 50 µg/mL and 117.11% at 100 µg/mL) were not significant compared to control. Higher oxidative stress was induced by MEL-NPs in B164A5 cells, the ROS production percentages gradually increasing to 103.30% at 10 µg/mL, 119.67% at 50 µg/mL and 129.5% at 100 µg/mL, without reaching statistical significance compared to control.

### 3.6 Influence of MEL-NPs on cell migration

**FIGURE 6:**
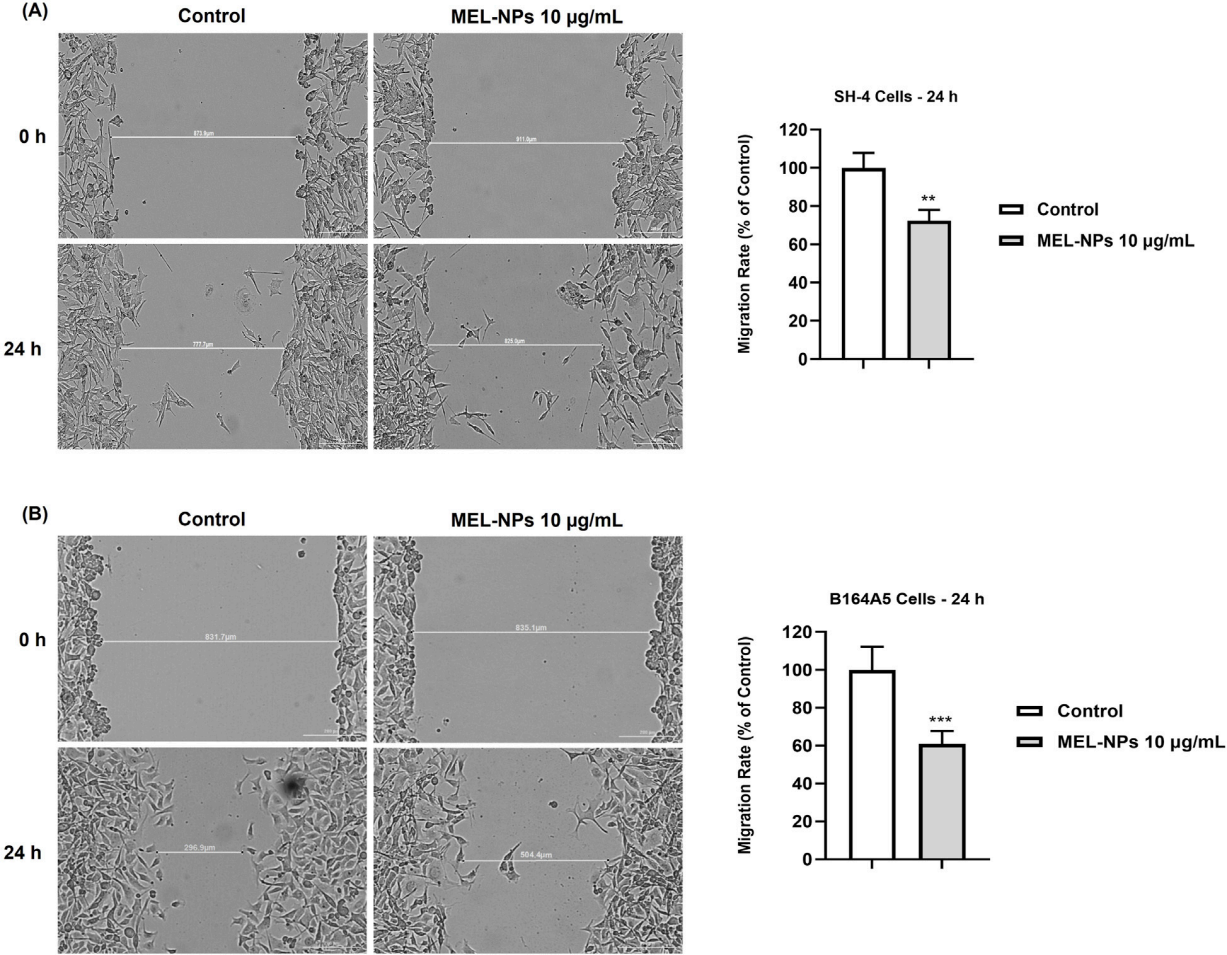
**(A)** Representative images illustrating the migratory capacity of SH-4 cells with and without the 24 h treatment with MEL-NPs 10 µg/mL and graphical representation of the impact of MEL-NPs 10 µg/mL on the migration rate of B164A5 cells after 24 h of treatment. **(B)** Representative images illustrating the migratory capacity of B164A5 cells with and without the 24 h treatment with MEL-NPs 10 µg/mL and graphical representation of the impact of MEL-NPs 10 µg/mL on the migration rate of B164A5 cells after 24 h of treatment. The data were normalized to control (representing cells without treatment) and expressed as means ± SD of three independent experiments conducted in triplicate. The statistical differences between Control and MEL-NPs-treated group were determined using the unpaired t-test (**p < 0.01; ***p < 0.001).

The influence of MEL-NPs after 24 h of stimulation on the migratory ability of CM cells was evaluated using the automatic scratch assay (Figure 6). The migratory ability of SH-4 cells was considerably inhibited by MEL-NPs at a low concentration (10 µg/mL), the calculated migration rate being 72.33% compared to control. A slightly higher motility blockage was obtained in the case of B164A5 CM cells, MEL-NPs 10 µg/mL significantly reducing the migration rate to 60.85%.

### 3.7 Effect of MEL-NPs on cell clonogenicity

**FIGURE 7:**
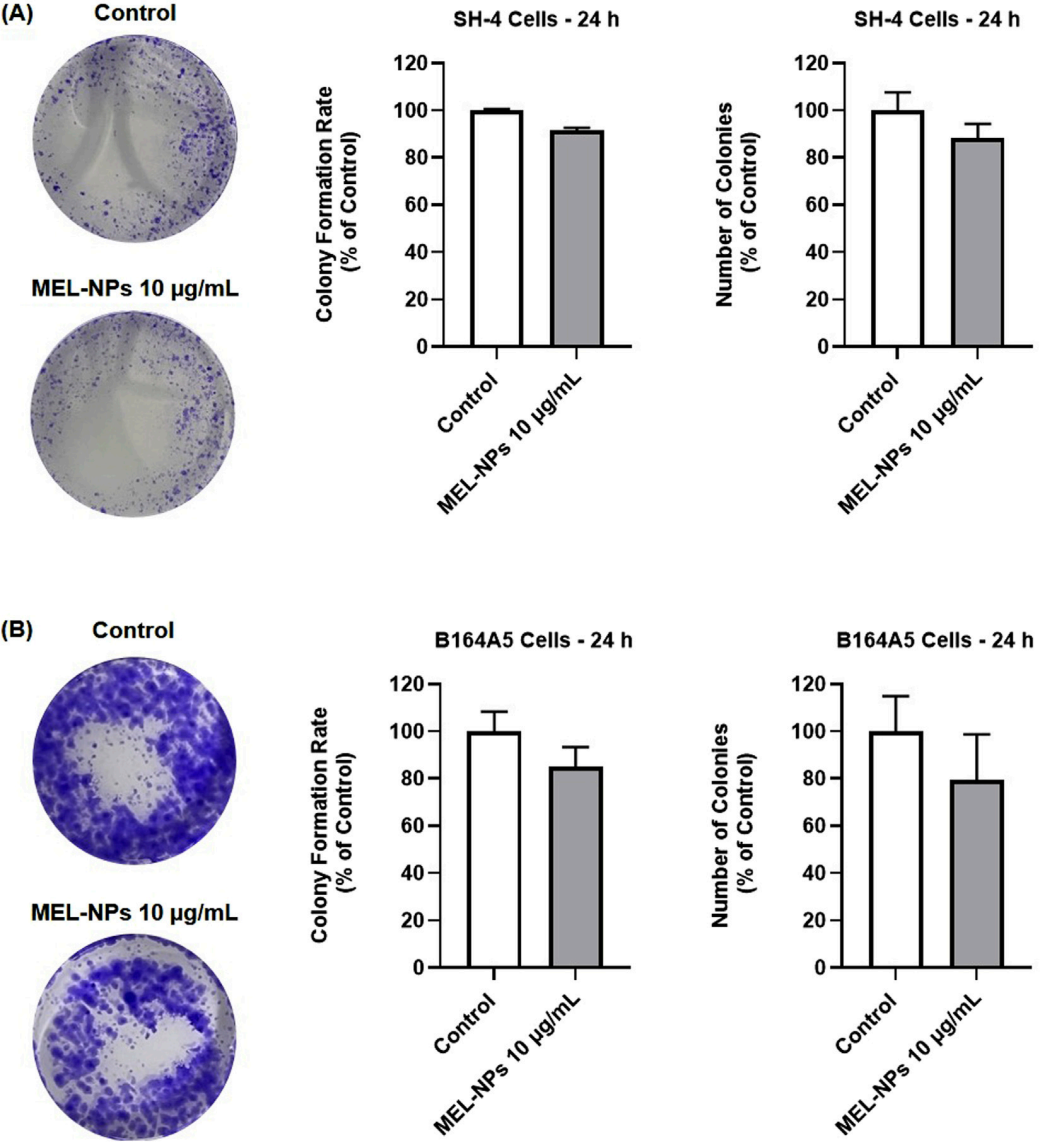
**(A)** Representative images of the crystal violet-stained SH-4 colonies with and without the 24 h treatment with MEL-NPs 10 µg/mL, as well as graphical representation of the impact of MEL-NPs 10 µg/mL on the colony formation rate and number of formed colonies in SH-4 cells after a 24 h treatment. **(B)** Representative images of the crystal violet-stained B164A5 colonies with and without the 24 h treatment with MEL-NPs 10 µg/mL, as well as graphical representation of the impact of MEL-NPs 10 µg/mL on the colony formation rate and number of formed colonies in B164A5 cells after a 24 h treatment. The data were normalized to control (representing cells without treatment) and expressed as means ± SD of three independent experiments conducted in triplicate. The statistical differences between Control and MEL-NPs-treated group were determined using the unpaired t-test.

The impact of MEL-NPs on the CM cells’ clonogenic properties was further investigated (Figure 7). At the concentration of 10 µg/mL, MEL-NPs suppressed the colony formation rate and reduced the number of formed colonies in SH-4 cells (to around 90%), however the effect was not significant compared to control. A slightly stronger inhibition in colony formation rate (to 85.13%) and reduction of colony number (to 79.44%) was obtained in B164A5 cells treated with MEL-NPs 10 µg/mL.

### 3.8 Impact of MEL-NPs on epithelial-to-mesenchymal transition (EMT) and apoptosis markers

**FIGURE 8:**
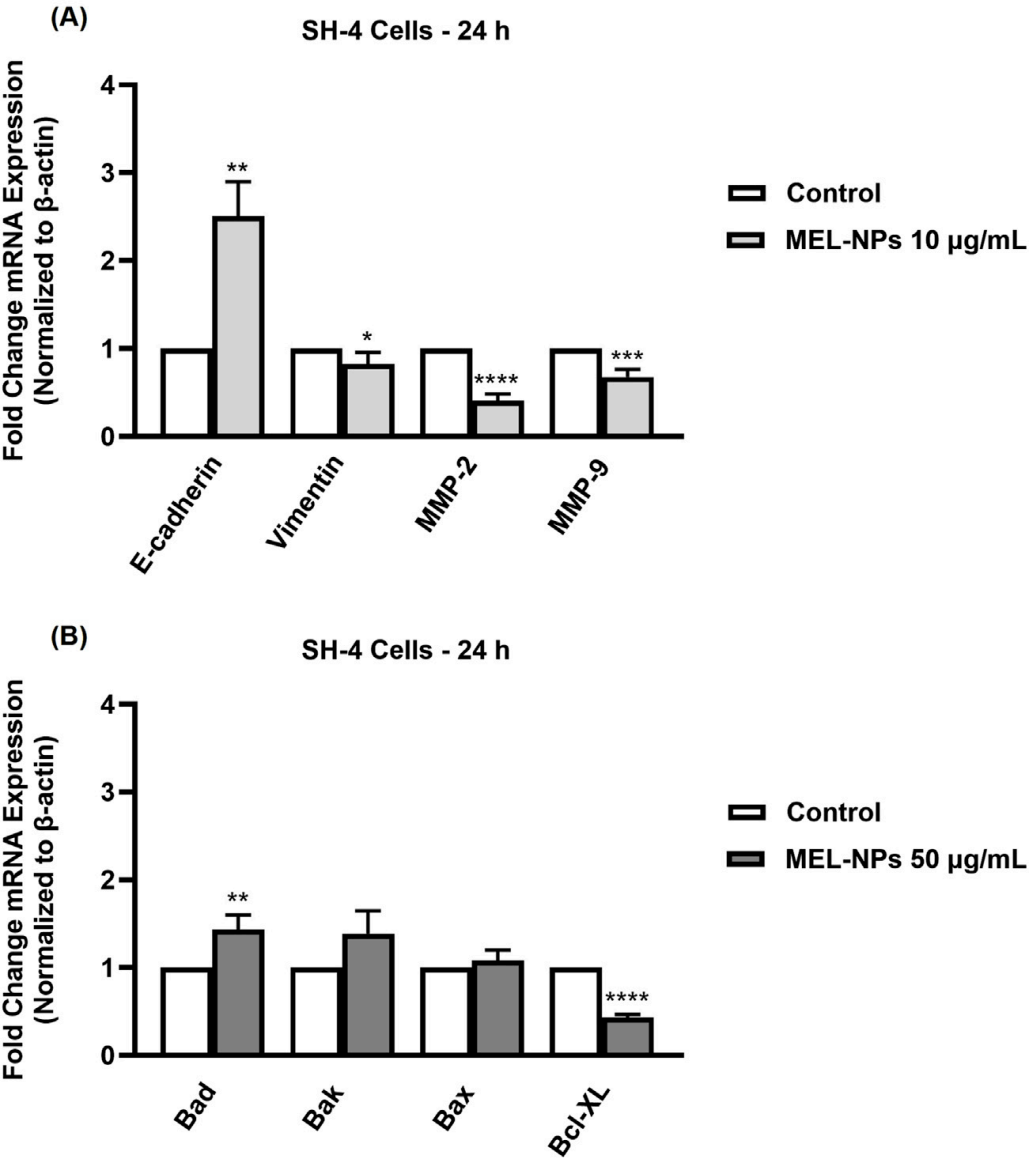
Relative fold change expression of mRNA expression (normalized to β-actin) of **(A)** EMT markers (E-cadherin, Vimentin, MMP-2, and MMP-9) and **(B)** apoptosis markers (Bad, Bak, Bax, Bcl-XL) in SH-4 cells after a 24 h treatment with MEL-NPs. The data were expressed as means ± SD of three independent experiments conducted in triplicate. The statistical differences between Control and MEL-NPs-treated group were determined using the unpaired t-test (*p < 0.05; **p < 0.01; ***p < 0.001; ****p < 0.0001).

The ability of MEL-NPs to target the EMT and apoptosis processes in SH-4 CM cells was assessed by determining their impact on specific markers (Figure 8). At the low concentration of 10 µg/mL, MEL-NPs caused a significant increase in the mRNA expression of E-cadherin, while also reducing vimentin, MMP-2, and MMP-9 expressions compared to control. At a higher concentration (50 µg/mL), MEL-NPs elevated the mRNA expression of the pro-apoptotic markers Bad, Bak, and Bax, but lowered the expression of the anti-apoptotic marker Bcl-XL. Statistical significance was reached only in the case of Bad and Bcl-XL.

### 3.9 Potential irritant effect and vascular toxicity of MEL-NPs

**FIGURE 9:**
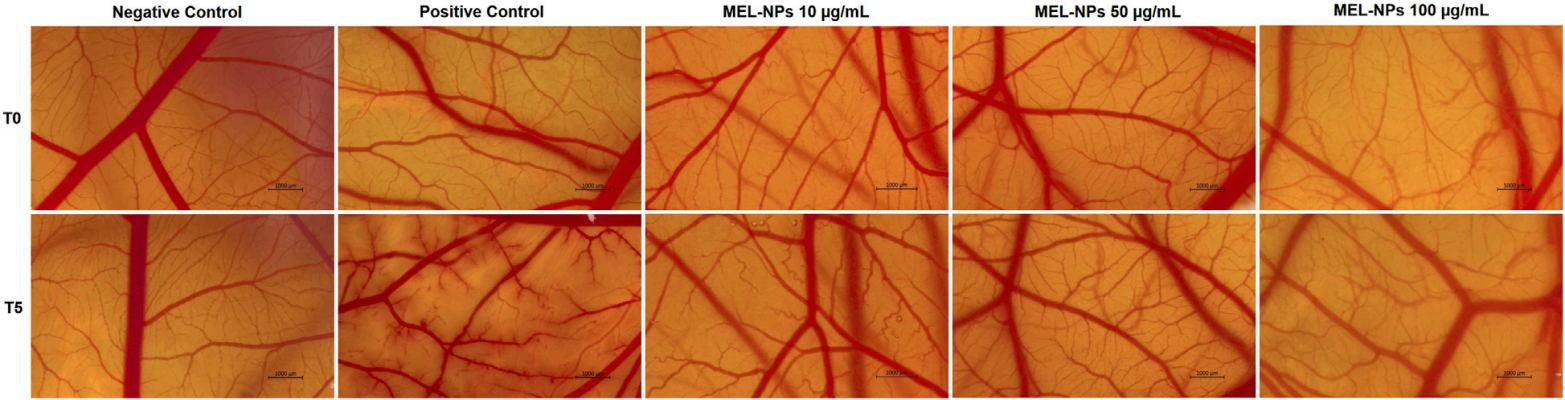
Stereomicroscopic images taken before the application of the evaluated samples (Negative Control–distilled water, Positive Control - Sodium Lauryl Sulfate 1%, and MEL-NPs 10, 50, and 100 µg/mL) on the chorioallantoic membrane (T0) and 5 min after their application (T5). The scale bars represent 1,000 µm.

The irritant potential of MEL-NPs 10, 50, and 100 µg/mL was evaluated *in ovo* by applying the HET-CAM test (Figure 9). Distilled water (H_2_O) used as negative control induced no alteration on the vascular structure, while SLS 1% (positive control) caused haemorrhage, lysis, and coagulation shortly after its application on the CAM. No severe vascular impairments were observed after the exposure of the CAM to MEL-NPs at the evaluated concentrations, except for slight signs of coagulation (at 50 µg/mL) and lysis (at 100 µg/mL) at the end of the treatments.

The calculated IS for the tested samples is presented in Table 2. The highest IS value was obtained for SLS 1%. MEL-NPs 10 µg/mL presented the same IS as H_2_O, while at higher concentrations (50 µg/mL and 100 µg/mL) it slightly increased to 0.34 and 0.49, respectively. MEL-NPs were classified as non-irritant on the CAM at these three concentrations, lacking vascular toxicity.

**TABLE 2:** Irritation score values for negative control (H_2_O), positive control (SLS 1%), and MEL-NPs (10, 50, and 100 µg/mL).

Sample	Irritation score (IS)	Irritant potential
Negative Control (H_2_O)	0.07	Non-irritant
Positive Control (SLS 1%)	19.88	Strong irritant
MEL-NPs 10 µg/mL	0.07	Non-irritant
MEL-NPs 50 µg/mL	0.34	Non-irritant
MEL-NPs 100 µg/mL	0.49	Non-irritant

### 3.10 Potential anti-angiogenic effect of MEL-NPs

**FIGURE 10:**
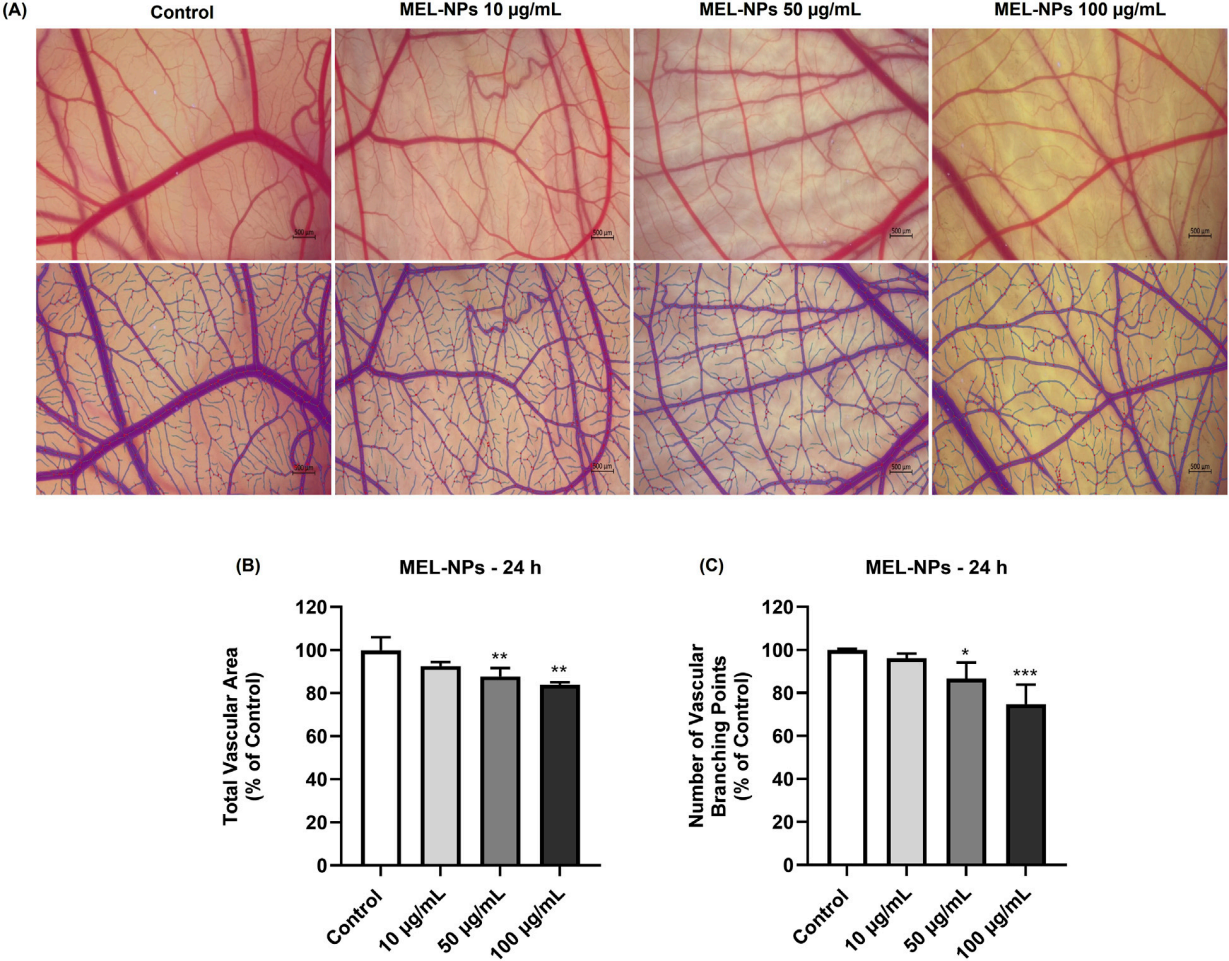
**(A)** Representative stereomicroscopic images (original and processed in IKOSA Prism) of the chorioallantoic membrane (CAM) vascularization after a 24 h treatment with MEL-NPs (10, 50, and 100 µg/mL). Control represents CAM without MEL-NPs treatment. The scale bars represent 500 μm. **(B)** Quantitative analysis of the total vascular area in the CAM treated for 24 h with MEL-NPs (10, 50, and 100 µg/mL). **(C)** Quantitative analysis of the number of vascular branching points in the CAM treated for 24 h with MEL-NPs (10, 50, and 100 µg/mL). The quantifications were performed using the IKOSA Prism AI Cam Assay (Version 3.1.0). The statistical differences between Control and the MEL-NPs-treated groups were calculated by one-way ANOVA analysis followed by Dunnett’s multiple comparisons post-test (*p < 0.05; **p < 0.01; ***p < 0.001).

Finally, the potential ability of MEL-NPs (10, 50, and 100 µg/mL) to inhibit neovascularization *in ovo* was evaluated. The results presented in Figure 10 indicate that the 24 h application of MEL-NPs on the CAM leads to a concentration-dependent reduction in the total vascular area, as well as in the number of vascular branching points, statistically significant changes being obtained at 50 and 100 µg/mL. At these concentrations, the total vascular area declined to 87.78% and 84%, respectively, while the number of vascular branching points was 86.65% and 74.78%, respectively.

## 4 Discussion

CM remains a leading contributor to the global increases in mortality rates caused by skin cancers, owing to its resistance to therapy, aggressive and metastatic nature, but also to the severe adverse effects and lack of tumor-specificity of the current treatment approaches (Cassano et al., 2021; Dhanyamraju and Patel, 2022). Thus, the modern pharmaceutical and biomedical research has prioritized the development of state-of-the-art site-specific therapeutics, the applications of nanotechnology promptly expanding within the area of CM diagnosis and treatment (Chen et al., 2013; Cassano et al., 2021). The outstanding properties and innate biocompatibility of MEL and PDA hastened the obtainment of MEL/PDA-based nanomaterials for cancer therapy (Qi et al., 2019; Zheng et al., 2022), being able to trigger therapeutic cellular responses *per se*, in the absence of drug cargoes (Liang et al., 2023). However, their applications in CM management lack a thorough investigation at present, requiring supplemental research. In the light of these data, the current work proposed the synthesis, physicochemical characterization, and preclinical pharmaco-toxicological screening of bioinspired MEL-NPs as a potential nanomedicine for CM, revealing several novelties, as follows: (i) the obtained MEL-NPs present a size of around 85.61 nm, a sphere-like shape, and a strong antioxidant potential; (ii) MEL-NPs retain a proper cytocompatibility in healthy skin cells (HEMa, HaCaT, and JB6 Cl 41-5a) at concentrations up to 75 µg/mL; (iii) MEL-NPs exert potent anti-tumor and anti-migratory activities in SH-4 and B164A5 CM cells after 24 h of treatment; (iv) the anti-neoplastic effect of MEL-NPs in CM cells is related to their efficacy in modulating the EMT, triggering apoptosis, and generating intracellular oxidative stress; and (v) MEL-NPs are non-irritant on the CAM and suppress angiogenesis *in ovo*.

The first point of interest in the current study was the physicochemical characterization of MEL-NPs (Figure 1) which were artificially produced through the controlled oxidative polymerization of dopamine in alkaline conditions, a method routinely employed for the obtainment of this type of nanoparticles which generally present a round morphology and a size up to 500 nm (Lemaster et al., 2019; Mavridi-Printezi et al., 2020; Marcovici et al., 2022). The as-synthesized MEL-NPs exhibited a clear sphere-like shape, and an average size of 85.61 nm with a small population of MEL-NPs presenting a dimension higher than 100 nm. The specific characteristics of their UV-VIS spectrum included a gradual decrease of absorbance from 200 nm to 1,000 nm, which is in accordance with the optical features of eumelanin (Ju et al., 2011), and other studies reporting the UV-VIS spectra of MEL or MEL-NPs (Li et al., 2018; Madkhali et al., 2019).

Oxidative stress plays a crucial role in different phases of melanomagenesis from tumor initiation and progression, to metastasis onset and chemoresistance development, CM exhibiting particularly elevated ROS levels compared to other solid cancers (Pizzimenti et al., 2021; Becker and Indra, 2023). Thus, antioxidants have become a promising strategy for CM management, with several radical-scavenging products showing potential applications in CM prevention and progression blockage (Becker and Indra, 2023). Based on these aspects, the study also included the assessment of the antioxidant activity retained by MEL-NPs at five increasing concentrations of interest – 10, 25, 50, 75, and 100 µg/mL, by applying the DPPH assay, defined as a simple, reliable, and widely used *in vitro* non-cellular test (Semenescu et al., 2023). MEL-NPs showed a concentration-dependent scavenging potential of the DPPH radical that was comparable to the antioxidant efficacy of ascorbic acid and gradually increased from 18.27% (at 10 µg/mL) to 84.17% (at 100 µg/mL), results that confirm previous reports, as MEL-like (PDA) nanostructures have been employed and thoroughly studied before as efficient oxidative stress scavengers (Yang et al., 2020). Lou et al. demonstrated the ROS-suppressing effects of PDA nanoparticles which showed a concentration-dependent clearance of O_2_−, OH, and DPPH radicals (Lou et al., 2021). Similarly, Ju and colleagues showed that the antioxidant activity of MEL-NPs increased dose-dependently up to 85%, stressing the importance of particle size in the DPPH scavenging ability of MEL-NPs: the smallest nanoparticles (68 ± 21 nm) exerting almost the same scavenging activity as ascorbic acid, while the ones with the highest size (291 ± 57 nm) presenting the lowest efficacy (Ju et al., 2011). The obtained results can be also correlated with the antioxidant ability of the natural pigment resulting from its efficacy in binding metal ions or quenching excited molecules and free radicals due to the chemical structure formed of phenol and indole groups, and especially to the eumelanin-specific DHICA unit that comprises of an extra carboxylate group (Caldas et al., 2020; Yang et al., 2020).

The second aspect investigated herein was the biocompatibility of MEL-NPs in cutaneous non-tumorigenic HEMa, HaCaT and JB6 Cl 41-5a cell lines, selected as representative *in vitro* models for cutaneous toxicity evaluation due to their specific characteristics. HEMa are human primary epidermal melanocytes presenting a needle-like, stellar, multipolar, or dendritic aspect and applications in skin diseases such as CM (https://www.atcc.org/). HaCaT represent spontaneously immortalized human keratinocytes (KCs) that contain similar surface markers and functionalities as isolated KCs and are employed in epidermal homeostasis and pathophysiology studies (Colombo et al., 2017; Blanchard et al., 2022). The murine JB6 Cl 41-5a epidermal cells exhibit an epithelial morphology and are currently employed in skin toxicity investigations (https://www.atcc.org/; Tewari-Singh et al., 2010). MEL-NPs are generally considered as highly biocompatible due to their similarity to the natural pigment which is produced within melanocytes in a nanosized form (Marcovici et al., 2022). The results obtained during this study (Figure 2) indicated that these nanoparticles triggered a concentration-dependent decline in the viability of skin-derived cells. Thus, MEL-NPs lacked cytotoxic potential in HEMa, HaCaT and JB6 Cl 41-5a cells up to the concentration of 75 µg/mL, while at 100 µg/mL a significant decline in cell viability (under 70%) was noticed. Moreover, at the lowest concentration, 10 µg/mL, MEL-NPs exerted a stimulatory effect on the viability of all healthy cell lines. In a recent report, PDA nanosheets presented a low cytotoxicity in L929 fibroblasts after 24, 72, and 144 h of treatment, the cells’ viability remaining over 90% (Chen et al., 2021). A significant decrease in the viability of mesenchymal stem cells’ viability was obtained only after their 24 h stimulation with MEL-NPs at a high concentration (200 µg/mL), while at lower ones (20 and 40 µg/mL), a stimulatory effect was noted (Tang et al., 2022).

The third interest of this research was the intrinsic anti-melanoma properties exerted by MEL-NPs that were explored using two melanogenic CM cell lines–SH-4 and B164A5. SH-4 is a human MEL-producing BRAF-mutated CM cell line formed of a mixture of spindle- and epithelial-shaped cells (https://www.atcc.org/; Bratu et al., 2020; Gambichler et al., 2023). B164A5 is a fibroblast-like highly-aggressive murine CM cell line capable of producing MEL in very high quantities (Danciu et al., 2013; Danciu et al., 2015), which was previously selected as an *in vitro* model for the investigation of the anti-tumor effects of various nanoplatforms (Soica et al., 2014; Danciu et al., 2019). According to Figure 2, MEL-NPs reduced the viability of SH-4 and B164A5 cells in a dose-dependent trend, a significant loss of viability being obtained starting with 25 µg/mL. Nonetheless, cytotoxicity, evidenced by a decline of viability under 70% according to the ISO Standard 10993-5:2009 (Marcovici et al., 2024), was obtained starting with the concentration of 50 µg/mL in CM cells compared to 100 µg/mL in healthy cutaneous cells. A previous study conducted by Nieto et al. described PDA as being an antineoplastic system by inducing cytopathic effects in BT4T4 and HCT116 cancer cells after 24, 48, and 72 h of treatment (Nieto et al., 2018), as well as to the findings of Perring and colleagues who described an increase in MO59K, RH30, RD, and U87 cancer cell death exposed to MEL-NPs for 24 h, at concentrations up to 200 µM (Perring et al., 2018). The cytocidal properties of MEL-NPs were associated with a significant shape change in SH-4 and B164A5 cells which became round and shrinked following treatment (Figure 3). One factor ensuring the successful application of nanomaterials in biomedicine is their interactions with target cells, resulting in cytoplasmic internalization and consequent cellular responses (Kim et al., 2012; Donahue et al., 2019). After 24 h of treatment, both SH-4 and B164A5 cells presented signs of pigmentation, evidencing the ability of MEL-NPs to enter and reside within their intracellular space and adopt a specific perinuclear localization. Nonetheless, a stronger pigmentation degree was noticed in B164A5 CM cells, indicating a potential more effective accumulation of MEL-NPs compared to the SH-4 cell line. The efficacy of the initial nanoparticle-cell interactions and cellular nanoparticle uptake depends on the properties of nanoparticles such as size, morphology, aggregation state, density, and sedimentation rate, but also on the cell phenotype and cell cycle phase (Kim et al., 2012; Donahue et al., 2019), which could partially explain the differences in the ability of SH-4 and B164A5 cells to internalize MEL-NPs. Moreover, as demonstrated in preceding studies, the PDA-NPs-cell interactions are facilitated by the dopamine receptors placed within the cell membrane and is performed through caveolae- and Rab34-mediated endocytosis, these nanoplatforms presenting an increased structural integrity within the intracellular space (Liang et al., 2023). Despite the observed differences in MEL-NPs uptake, their cytotoxic potential was similar in both SH-4 and B164A5 CM cells. The potential cell death behind the anti-CM properties of MEL-NPs was another aspect of interest for this study. Considering that apoptosis is distinguished from other cell death types due to its unique morphological features such as cell shrinkage and blebbing, nuclear constriction, cleavage of chromatin into pyknotic bodies, actinomyosin ring contraction and microtubule reorganization (Povea-Cabello et al., 2017; Yan et al., 2020), the aspect of the nuclei and cytoskeletal filaments in the CM cells treated with MEL-NPs was explored. These nanoparticles were found to produce several apoptotic-like alterations in cell nuclei indicated by a visible size reduction, and a massive chromatin condensation at all tested concentrations, and changes in cytoskeletal dynamics such as tubulin and F-actin constriction associated with cell dysmorphology, rounding and blebbing (Figure 4). The study continued with an assessment of the potential ability of MEL-NPs to trigger oxidative stress in SH-4 and B164A5 CM cells considering that redox homeostasis targeting was described as an appealing therapeutic strategy for CM (Becker and Indra, 2023) and that one potential mechanism underlying the anti-tumor activity of PDA nanoparticles is ROS production (Nieto et al., 2018). The findings presented in Figure 5 indicated an increased but statistically non-significant level of ROS in both CM cell lines treated with MEL-NPs (10, 50 and 100 µg/mL) for 24 h, with higher oxidative stress being caused in B164A5 cells, which can be related to the more pronounced internalization of MEL-NPs in this cell line compared to SH-4 cells as illustrated in Figure 3. The observed double-edge properties of these nanostructures that, on one hand scavenge oxidants (Figure 1E) while on the other hand trigger oxidative stress (Figure 5), were previously explained by Liu et al. who found that PDA is redox-active and has electron-donating abilities, being able to donate electrons not only to O_2_ to generate ROS, but also to free radicals to quench them (Liu et al., 2019).

At the lowest non-cytotoxic concentration of 10 µg/mL, MEL-NPs significantly suppressed the motility of both SH-4 and B164A5 CM cells, causing a reduction in migration rate following treatment (Figure 6). In a previous study, it was found that PDA-NPs associated with a mild photothermal effect hindered the migration of CM and breast cancer cells without causing cell death. The observed effects were associated with the ability of PDA-NPs to efficiently target the transmembrane MUC18 marker highly expressed in metastatic cancers and subsequently alter cytoskeletal actin dynamics, as well as cell morphology and stiffness (Liu et al., 2021). The impaired SH-4 and B164A5 cell migratory ability caused by MEL-NPs 10 µg/mL was also accompanied by repressed clonogenic properties and inhibited colonization (Figure 7), a process described as micrometastasis formation (Werner-Klein et al., 2018), although the inhibitions were not significant compared to control. The EMT is a complex biologic process through which tumor cells lose their epithelial character, acquire a mesenchymal phenotype and consequently gain enhanced migratory and invasive capacity along with elevated resistance to apoptosis and therapy (Pedri et al., 2022). EMT is characterized by an increase in the expression of mesenchymal proteins such as vimentin and matrix metalloproteinases (MMPs), as well as a loss in the expression of proteins involved in epithelial integrity maintenance (e.g., E-cadherin, Occludins, Claudins, etc.) (Pearlman et al., 2017; Dongre and Weinberg, 2019). Its contribution to melanoma aggressiveness has been extensively documented up to date, therefore becoming a potential therapeutic target for diminishing its invasion and metastasis formation abilities (Pearlman et al., 2017). As presented in Figure 8, after a 24 h treatment of SH-4 CM cells, MEL-NPs 10 µg/mL modulated the mRNA expressions of several markers involved in EMT by significantly elevating E-cadherin and inhibiting vimentin, MMP-2 and MMP-9. E-cadherin represents a well-studied type-I classical cadherin and a strong tumor suppressor which plays a crucial role in maintaining the cell epithelial phenotype (Loh et al., 2019), its loss of expression in melanoma cells being associated with increased proliferation and mobility (D’arcy and Kiel, 2021). Vimentin is a key EMT biomarker present in mesenchymal cells and usually overexpressed during cancer metastasis (Usman et al., 2021), while MMP-2 and MMP-9 are peptidases involved in extracellular matrix remodeling and tumor invasive processes, favoring melanoma spreading and metastasis (Napoli et al., 2020). A recently published study reported the development of N-cadherin targeted MEL-NPs able to reverse the EMT in vascular endothelial cells and thus potentially slow cancer progression (Liu et al., 2024). According to the results from Figure 8, at a high and cytotoxic concentration (50 µg/mL), MEL-NPs were also found to trigger apoptosis by increasing pro-apoptotic Bad, Bak, and Bax and suppressing anti-apoptotic Bcl-XL, specific biomarkers belonging to the B-cell lymphoma 2 (Bcl-2) superfamily of proteins that regulates the intrinsic mitochondrial apoptosis pathway and is highly dysfunctional in different human cancers, including melanoma (Coricovac et al., 2021). However, statistical significance was obtained only in the case of Bad and Bcl-XL markers. This apoptosis-inducing effect of MEL-NPs relates to previous findings on the cell death mechanisms of MEL in cancer cells. For instance, Al-Obeed et al. previously showed the pro-apoptotic effect of herbal MEL in colorectal carcinoma cells which was associated with increased cytochrome c release, inhibition of Bcl-2 proteins and Caspase −3/−7 activation (Al-Obeed et al., 2020).

The last point of interest in the pharmaco-toxicological screening of MEL-NPs was the *in ovo* evaluation of their potential vascular toxicity and angio-inhibitory effect using the CAM, a vascularized extraembryonic membrane with versatile applications in the evaluation of nanomaterials in terms of mucosal irritant potential and impact on vascularization, among others (Buhr et al., 2020). As presented in Figure 9 and Table 2, at the concentrations of 10, 50, and 100 µg/mL, MEL-NPs lacked irritant activity on the CAM, inducing no significant alterations in the structure of the blood vessels and presenting an IS score lower than 0.9. Angiogenesis, the complex process leading to the formation of new blood vessels, is crucial for the occurrence and development of CM that requires a sustained supply of nutrients and oxygen to grow. Therefore, angiogenesis inhibition using anti-angiogenic drugs has emerged as a potential therapeutic measure for CM (Wu et al., 2022a). As highlighted in Figure 10, MEL-NPs exerted an angiostatic effect *in ovo* by suppressing CAM neovascularization, significant reductions in the total vascular area (to 87.78% and 84%) and number of vascular branching points (to 86.65% and 74.78%) being obtained at 50 and 100 µg/mL. By investigating similar concentrations as the ones tested herein, namely 25, 50, and 100 µg/mL, a recent study has demonstrated the ability of PDA-PEG nanoparticles to inhibit osteoclast-related angiogenesis, and block blood vessel formation *in vivo* (Wu et al., 2022b).

Although the present study provided novel perspectives on the innate anti-tumor properties of MEL-NPs describing their promising application in CM treatment, some limitations should be considered in future studies. On one hand, the pro-apoptotic effect of MEL-NPs in CM cells was assessed by evaluating the specific hallmarks of apoptosis such as nuclear and cytoskeletal morphological changes and the expression of apoptosis markers, while additional methods are necessary to fully capture the complexity of the cell death underlying the observed effects of MEL-NPs in CM cells. On the other hand, the full understanding of the potential utilization of MEL-NPs in CM treatment in terms of safety and efficacy should be further extended by validating these *in vitro* and *in ovo* findings through *in vivo* studies.

## 5 Conclusion

Built upon previous contributions, this study furthers the current understanding on the anti-tumor activity of MEL-NPs and their utilization in cancer therapy, opening a new avenue for the therapeutic approach of CM by redirecting the applications of MEL-NPs towards the management of skin malignancies. The findings related herein show that MEL-NPs selectively target CM cells, while also presenting a proper, but concentration-dependent cytocompatibility in healthy cutaneous cells, illustrate the specific intracellular localization of MEL-NPs in the perinuclear area of CM cell lines, and demonstrate the innate anti-melanoma activity of MEL-NPs which was correlated to their ability to trigger apoptosis-related cytotoxicity, cause oxidative stress and block cell motility by targeting the EMT. The conducted *in ovo* screenings also classified MEL-NPs as a non-irritant nanoplatform with angio-inhibitory properties.

## Data Availability

The original contributions presented in the study are included in the article/supplementary material, further inquiries can be directed to the corresponding author.
